# Analysis of Clinical and Histopathological Findings in Microscopic Colitis

**DOI:** 10.5146/tjpath.2022.01574

**Published:** 2022-09-15

**Authors:** Gozde Topel, Ebru Cakır, Ilgin Aydın, Fatma Husniye Dılek, Aysegul Akder Sarı

**Affiliations:** Department of Pathology, Izmir Katip Celebi University, Ataturk Training and Research Hospital, Izmir, Turkey

**Keywords:** Colitis, Microscopic colitis, Collagenous colitis, Lymphocytic colitis

## Abstract

***Objective:*** Microscopic colitis is a chronic inflammatory disorder characterized by a triad of chronic diarrhea, endoscopy without significant abnormality, and distinct histopathological features. Histopathologically, microscopic colitis is divided into 3 subtypes; collagenous colitis, lymphocytic colitis, incomplete microscopic colitis. The main purpose of this study was to analyze the detailed clinicopathological parameters of microscopic colitis cases in the Turkish population.

***Material and Method:*** The clinicopathological parameters were evaluated in 53 microscopic colitis cases (37 collagenous colitis, 7 lymphocytic colitis, 9 incomplete microscopic colitis) diagnosed between 2010 and 2019.

***Results:*** All cases had lymphoplasmacytosis. The presence of ≥20 eosinophils/high power field in the lamina propria was remarkable in 75.7%, 57.1%, and 11.1% of collagenous colitis, lymphocytic colitis, and incomplete microscopic colitis cases, respectively. One of the striking findings was the presence of concomitant Celiac disease in 29% of the lymphocytic colitis cases. In terms of drug use, proton pump inhibitors and nonsteroidal anti-inflammatory drugs were the most commonly used drugs.

***Conclusion:*** The mean age in our series is lower than the literature and a distinct male predominance was observed in lymphocytic colitis and incomplete microscopic colitis, contrary to the literature. These suggest that susceptibility to microscopic colitis may differ between ethnic groups. The presence of overt lymphoplasmacytosis, eosinophilic infiltration and epithelial damage are the microscopic features which should alert the pathologist for the diagnosis of complete microscopic colitis. Given that microscopic colitis is a common treatable cause of chronic diarrhea, awareness of the aforementioned histopathological features is of utmost importance for accurate diagnosis and not to miss incomplete cases.

## INTRODUCTION

Microscopic colitis (MC) is a chronic inflammatory bowel disorder characterized by a triad of chronic diarrhea, endoscopy without significant abnormality, and distinct histopathological features (1). MC has been considered to be a rare disease but currently it is diagnosed in about 10% of patients investigated by colonoscopy for chronic diarrhea (2,3). There is a female predominance with a mean age of 65 years at diagnosis. Female preponderance appears to be more pronounced in collagenous colitis compared to lymphocytic colitis (female-to-male incidence rate ratio 3.0 and 1.9, respectively) (4). The exact etiology of MC is unknown, but it is most likely multifactorial, and infection, autoimmunity, smoking, drugs, mucosal immunopathology, dysregulated collagen metabolism, and genetics have been thought to play a role in the etiology (5–7). Three histological subtypes have been defined: collagenous colitis (CC), lymphocytic colitis (LC), and incomplete microscopic colitis (MCi) (1,8,9). Lymphocytic colitis is described by an increased number of surface intraepithelial lymphocytes (>20 intraepithelial lymphocytes (IELs)/100 enterocytes), and collagenous colitis by a thickened collagen band (>10 μm) below the surface epithelium (1,10). There is increased mixed inflammation in the lamina propria and surface epithelial damage but only little crypt architectural distortion, if any (1,11). Incomplete and variant forms disclosing less characteristic features have been described by many different names such as borderline LC, minimal CC, microscopic colitis not otherwise specified (MCnos), paucicellular lymphocytic colitis, and MCi (1). The differential diagnosis includes ulcerative colitis and Crohn’s disease, infectious colitis, diverticular disease, and amyloidosis in particular (12). It may not be easy to distinguish between non-specific inflammatory mucosal changes and MC, especially the incomplete forms (9,13). Some patients with MC go into spontaneous remission. Although antidiarrheals such as loperamide, diphenoxylate or bismuth subsalicylate are used, the most promising evidence-based therapeutic option is currently budesonide, a locally active corticosteroid (8). Relapse following treatment has been reported to be common in controlled trials; however, the long-term course of MC and especially MCi is not well-known (9,14). Since incomplete forms are reported to progress to MC in %30 of follow-up biopsies, it is important to recognize these cases (15). There are only few studies in the English literature on histomorphological features of MC and MCi in detail. The main purpose of this study was to analyze the detailed histopathological and clinical parameters of MC and MCi cases in the Turkish population.

## MATERIALS and METHODS

### Patients

The histopathological parameters were re-evaluated in 53 microscopic colitis cases (37 CC, 7 LC, 9 MCi) diagnosed between January 2010 and December 2019. The study protocol was approved by the institutional ethics committee on January 21, 2021 (decision no: 0008). Clinical and demographic data were retrieved from the hospital records.

### Histopathological Information

**Figure 1:**
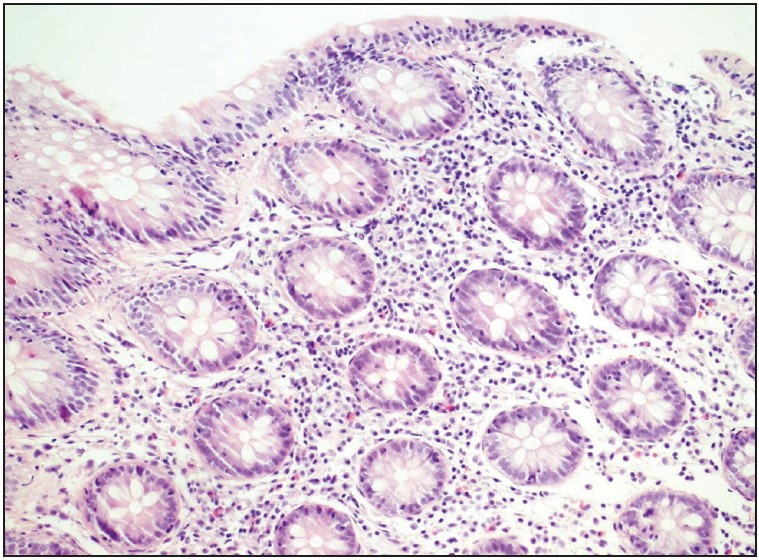
Increased IEL/100 enterocytes, moderate lymphoplasmocytosis and mild epithelial damage characterised as mucin loss in LC case (H&E, x100).

**Figure 2:**
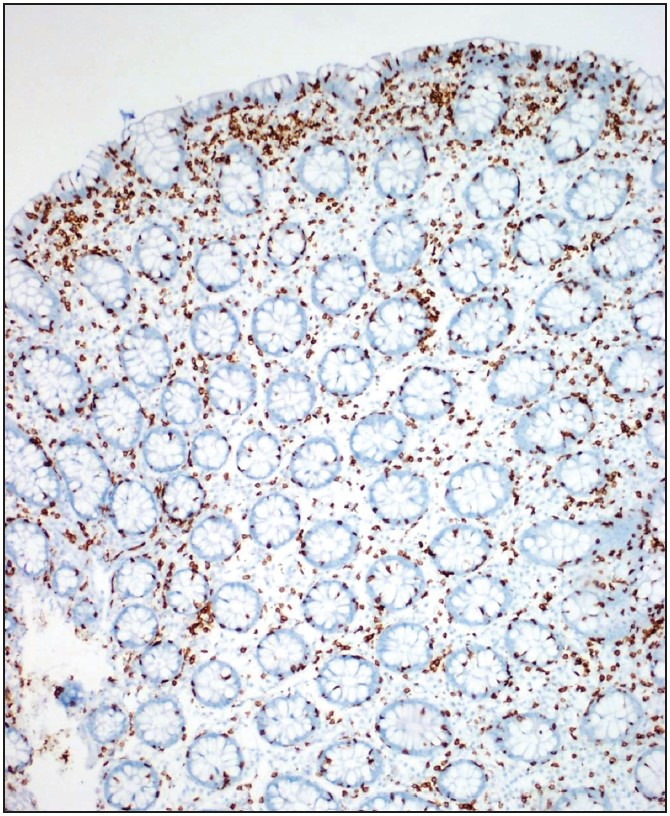
CD3 stain highlights increased IEL/100 enterocytes (x100).

We retrospectively reviewed all colon biopsies for each patient and noted the biopsy indication and symptoms, associated diseases, and the location and number of biopsies obtained. Histopathological reviews were based on hematoxylin and eosin (H&E) staining. In borderline cases, CD3 immunostaining to evaluate the number of intraepithelial lymphoctes and Masson Trichrome (MT) to evaluate collagen density were also available for review. All slides were re-evaluated by 2 pathologists; the presence of a subepithelial collagen band >10 μm in thickness and >20 intraepithelial lymphocytes (IELs)/100 enterocytes were considered diagnostic for CC and LC, respectively. The presence of a subepithelial collagen band 5-10 μm in thickness and/or 10-20 IELs/100 enterocytes were classified as MCi (10) (Figure 1 and Figure 2).

**Figure 3:**
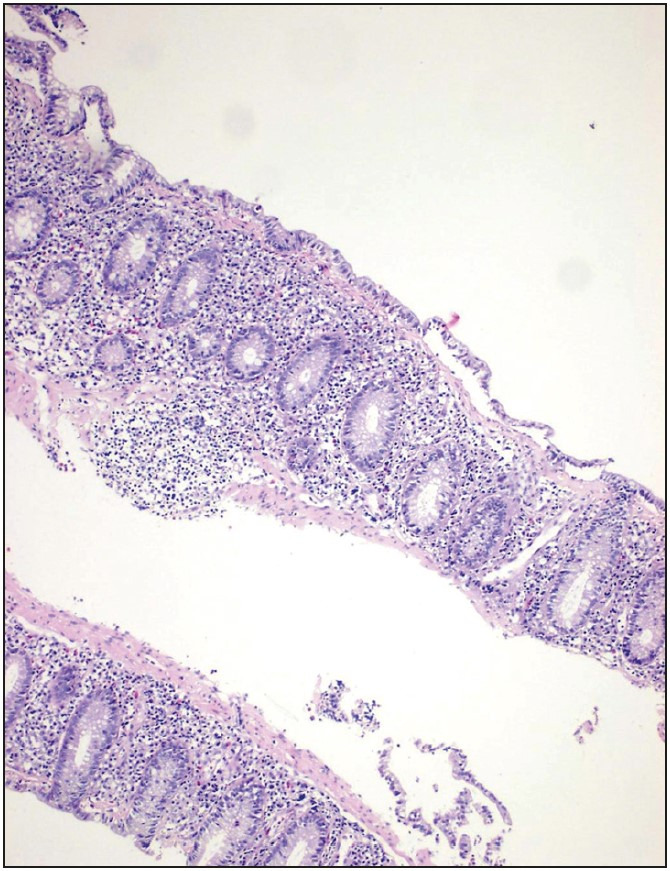
Epithelial damage with sloughing of the superficial mucosa and increased subepithelial collagen band thickness in CC case (H&E, x100).

Detailed histopathological examination for the following parameters were carried out: the collagen band thickness, presence of neutrophils, lymphoplasmacytes and eosinophils in the lamina propria, intraepithelial lymphocyte count, presence of crypt distortion, crypt abscess, giant cell, granuloma and paneth cell metaplasia, entrapment of erythrocytes and other cells in the subepithelial collagen band, the evidence of the epithelial damage as mucin depletion, vacuolization, sloughing of the superficial mucosa and detachment of the surface epithelium. Inflammatory infiltrate within the lamina propria was scored as follows: lymphoplasmacytic intensity was graded as mild and moderate/severe and the location was categorized as superficial or superficial/deep. Entrapment of cells was graded as slight/moderate/severe. The number of eosinophils in the lamina propria was grouped as 0-19, 20-49 and ≥50/HPF. Intraepithelial lymphocyte count was grouped as 0-4, 5-9, 10-20, and >20/100 enterocytes. The presence of IELs was evaluated in areas without lymphoid aggregates, which are frequent in the colon and are associated with increased IELs that would not in any way suggest lymphocytic colitis/microscopic colitis. Subepithelial collagen band thickness measurement was performed using the tools section of the application (Olympus Labsens). Special care was taken to avoid misinterpretation of a tangentially cut basement membrane while measuring the collagen band thickness. Biopsy sites were categorized as ascending colon, transverse colon, descending colon and rectosigmoid colon. Multiple endoscopic biopsies taken from 2-4 sites from the patients were evaluated. Biopsies from the ceacum, ascending colon, and transverse colon were classified as right-sided and biopsies from the descending colon and rectosigmoid as left-sided. The clinic data such as initial symptoms, the association of other disease (celiac disease, helicobacter pylori gastritis, gastrointestinal neoplasia), and drug history were recorded (Figure 3).

### Data Analysis

Statistically, differences between groups for the categorical clinicopathological variables were analyzed by the χ2 test, using both Fisher's Exact test and the Fisher-Freeman-Halton Exact test (SPSS version 22, IBM SPSS Inc., Armonk, NY, USA). P*<*0.05 was considered statistically significant.

## RESULTS

### Patient Demographics

A total of 53 cases with the diagnosis of microscopic colitis were included in the study.

**Figure 4:**
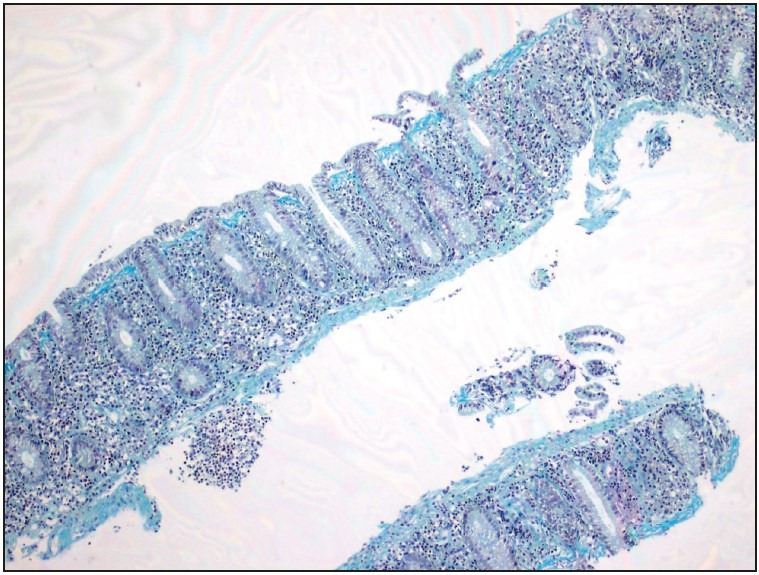
Masson Trichrome stain highlights increased subepithelial collagen band thickness (x100).

Fifty one of the cases were diagnosed with microscopic colitis in the initial biopsy, one case in the 3rd biopsy, and the remaining case in the 4th biopsy. Thirty seven (70%) cases were CC, seven (13%) cases were LC, and nine (17%) cases were MCi with a mean age of 59 (22-87), 51 (27-83) and 46 (24-72) years respectively. When patients were divided into two groups according to age as ≤50 years old and >50 years old, 73.0% of CCs, 42.9% of LCs, and only 33.3% of MCi were found to be over 50 years of age. Patients with CC were statistically significantly older (most were >50 years of age) when compared to lymphocytic colitis and incomplete microscopic colitis cases (p=0.045). CC had a female predominance (n=26, 70.3%), while there was a male predominance in LC (n=7, 100%) and MCi (n=7, 77.8%). The gender difference between CC and non-CC cases was statistically significant (p<0.001). The concomitant disease and drug use history in MC cases are summarized in Table 1 and the clinical features of MC and MCi cases are summarized in Table 2 and Table 3 (Figure 4).

Biopsies were taken from 5 different sites in 15, 4 in 9, 3 in 19, and 2 different sites in 10 of 53 cases. The frequency order of biopsy sites was the transverse colon, ceacum-ascending colon, rectosigmoid colon and descending colon, respectively. There was a predominance of right colonic localization in 60% of CC and 77% of LC and left colonic involvement in 60% of MCi cases. There were multiple biopsy specimens in each case, and the distribution according to all biopsy localizations was as follows; in CC: ceacum - ascending colon (39%), transverse colon (24%), descending colon (18%) and rectosigmoid colon (19%); and in LC: transverse colon (44%), ceacum - ascending colon (33%), with equal rates in the descending colon and rectosigmoid colon (11%); and in MCi: rectosigmoid colon (60%), descending colon (30%), transverse colon (10%). All patients presented with chronic watery diarrhea. Concomitant diseases were; Helicobacter pylori gastritis (n=7, 13%), Celiac disease (n=3, 6%), inflammatory bowel disease (n=3, 6%; 2 cases were defined as ulcerative colitis), autoimmune diseases (Hashimoto thyroiditis (n=2, 4%); Sjogren disease (n=1, 2%); rheumatoid arthritis n=4, (8%); Behçet disease (n=1, 2%)), coronary artery disease and hypertension (n=12, 23%), diabetes mellitus (n=6, 11%). Strikingly, 29% of LC cases had concomitant Celiac disease. In terms of drug use history, PPIs (n=9, 17%) and NSAIDs (n=8, 15%) were the most common medications. SSRI and colchicine use was noted in one patient (2%). Notably, 8 out of 9 patients with a history of PPI use had CC (Table 1).

**Table 1:** Concomitant disease and drug use history in MC cases.

**Concomitant disease**	**Microscopic Colitis, n (%)**
HP Gastritis	7 (13)
Celiac Disease	3 (6)
Inflammatory Bowel Disease	3 (6)
Hashimoto Thyroiditis	2 (4)
Rheumatoid Arthritis	4 (8)
Behçet Disease	1 (2)
Sjogren Disease	1 (2)
Coronary artery disease and Hypertension	12 (23)
Type 2 Diabetes Mellitus	6 (11)
**Drug usage**	
PPI	9 (17)
NSAIDs	8 (15)
Colchicine	1 (2)
SSRI	1 (2)

*HP: Helicobacter pylori, **PPI:** Proton pump inhibitor, **NSAIDs:** Nonsteroidal anti-inflammatory drugs, **SSRI:** Selective Serotonine Reuptake Inhibitor.

### Histopathological Findings

The detailed histopathological features of MC and MCi cases are summarized in Table 2 and Table 3. All cases had lymphoplasmacytosis in the lamina propria which was mild in 45.9% (17/37), 86% (6/7), 100% (9/9) of CC, LC and MCi, respectively. Moderate/severe lymphoplasmacytosis was present in more than half of CC (54.1%) while it was observed in only 14% of LC and in none of the MCi. The difference between the CC and non-CC cases was statistically significant (p=0.002).

**Table 2:** Clinical and histopathological features of MC and MCi cases.

	**Collagenous Colitis** **n=37 (%)**	**Lymphocytic Colitis** **n=7 (%)**	**Incomplete Microscopic Colitis** **n=9 (%)**	**p**
**Age** ≤50 >50	10 (27.0) 27 (73.0)	4 (57.1) 3 (42.9)	6 (66.7) 3 (33.3)	0.045
**Sex** Female Male	26 (70.3) 11 (29.7)	0 (0) 7 (100)	2 (22.2) 7 (77.8)	<0.001
**IELs/100 enterocytes** 0-4 5-9 10-20 >20	1 (2.7) 21 (56.8) 14 (37.8) 1 (2.7)	0 (0) 0 (0) 0 (0) 7 (100)	3 (33.3) 5 (55.6) 1 (11.1) 0 (0)	<0.001
**Collagen band (μm)** <30 ≥30	27 (73.0) 10 (27.0)	7 (100) 0 (0)	9 (100) 0 (0)	0.080
**Lymphoplasmocytosis in lamina propria** Slight Moderate/severe	17 (45.9) 20 (54.1)	6 (86.0) 1 (14.0)	9 (100) 0 (0)	0.003
**Eosinophils/1HPF in lamina propria** 0-19 20-49 ≥50	9 (24.3) 21 (56.8) 7 (18.9)	3 (42.9) 4 (57.1) 0 (0)	8 (88.9) 1 (11.1) 0 (0)	0.008
**Surface epithelial damage** Sloughing Flattening Vacuolization Loss of mucin	27 (73.0) 19 (51.4) 24 (65.0) 23 (62.2)	2 (29.0) 6 (86.0) 3 (43.0) 5 (71.0)	3 (33.0) 1 (11.0) 2 (22.0) 3 (33.0)	0.014 0.127 0.012 0.087
**Entrapped erythrocytes in subepithelial collagen**	34 (91.9)	4 (57.1)	5 (55.6)	0.006
**Distinct entrapped cells in subepithelial collagen**	20 (54.1)	1 (14.3)	0 (0)	0.003
**Neutrophil leukocytes forming cryptitis**	3 (8.1)	0 (0)	0 (0)	1.000
**Paneth cell metaplasia**	6 (16.2)	0 (0)	0 (0)	0.411

*IELs: Intraepithelial lymphocytes, *HPF: High power field.

**Table 3:** Clinical and histopathological features of collagenous colitis and non-collagenous colitis cases.

	**Collagenous Colitis** **n=37 (%)**	**Non-collagenous Colitis** **n=16 (%)**	**p**
**Age** ≤50 >50	10 (27) 27 (73)	10 (62.5) 6 (37.5)	0.029
**Sex** Female Male	26 (70.3) 11 (29.7)	2 (12.5) 14 (87.5)	<0.001
**IELs/100 enterocytes** 0-4 5-9 10-20 >20	1 (2.7) 21 (56.8) 14 (37.8) 1 (2.7)	3 (18.8) 5 (31.3) 1 (6.3) 7 (43.8)	<0.001
**Collagen band (μm)** <30 ≥30	27 (73.0) 10 (27.0)	16 (100) 0 (0)	0.021
**Lymphoplasmocytosis in lamina propria** Slight Moderate/severe	17 (45.9) 20 (54.1)	15 (93.8) 1 (6.3)	0.002
**Eosinophils/1HPF in lamina propria** 0-19 20-49 ≥50	9 (24.3) 21 (56.8) 7 (18.9)	11 (68.8) 5 (31.3) 0 (0)	0.006
**Surface epithelial damage** Sloughing Flattening Vacuolization Loss of mucin	27 (73.0) 19 (51.4) 24 (65.0) 23 (62.2)	5 (31.2) 7 (43.7) 5 (31.2) 8 (50.0)	0.006 0.743 0.008 0.214
**Entrapped erythrocytes in subepithelial collagen**	34 (91.9)	9 (56.3)	0.002
**Distinct entrapped cells in subepithelial collagen**	20 (54.1)	1 (6.3)	0.001
**Neutrophil leukocytes forming cryptitis**	3 (8.1)	0 (0)	0.241
**Paneth cell metaplasia**	6 (16.2)	0 (0)	0.087

*IELs: Intraepithelial lymphocytes, *HPF: High power field

The majority of CC (n=36, 97.3%) and MCi (n=6, 66.7%) showed 5-20 IELs/100 enterocytes. All LC cases showed >20 IELs/100 enterocytes (mean 33 IELs/100 enterocytes). In 97.3% of CCs, IEL was seen in the range of 5-20/100 enterocytes and in 37.8% in the range of 10-20/100 enterocytes. Intraepithelial lymphocyte count was in the range of 10-19/100 enterocytes and the subepithelial collagen band was below 5 μm in one of the MCi cases in which we described as lymphocytic type of incomplete microscopic colitis. Intraepithelial lymphocyte count was <10 in the other 8 cases that exhibited subepithelial collagen band 5-10 μm in thickness and these were interpreted as collagenous type MCi.

One of the remarkable findings was the presence of ≥20 eosinophils/1HPF in the lamina propria in 75.7% (28/37), 57.1% (4/7), 11.1% (1/9) of CC, LC, MCi, respectively and the difference was statistically significant (p=0.008).

Sloughing of the superficial mucosa, flattening, vacuolization, and loss of mucin in the surface epithelium were identified in 73% (27/37), 51.4% (19/37), 65% (24/37), 62.2% (23/39) of CC; 29% (2/7), 86% (6/7), 43% (3/7), 71% (5/7) of LC while 33% (3/9), 11% (1/9), 22% (2/9), 33% (3/9) of MCi, respectively. Epithelial damage with sloughing of the superficial mucosa in CCs was significantly more frequent than in lymphocytic and incomplete microscopic colitis (p=0.014). Epithelial damage characterized as vacuolization in MCi was lower than in CC and LC cases (p= 0.012).

Paneth cell metaplasia was observed in only CCs (n=6/37) (not in LC or MCi). Crypt distortion, crypt abscess, giant cell, granuloma, and ulceration were not identified in any of the cases. Neutrophil leukocytes forming cryptitis were present in only 3 of 37 (8.1%) CC cases, and in none of the LC and MCi cases.

Entrapped erythrocytes and cells other than erythrocytes in the subepithelial collagen band was observed in 91.9% (34/37) and 54.1% (20/37) of CCs, respectively, but they were significantly less common features for LC cases (57.1% (4/7), 14.3% (1/7)) and MCi cases (55.6% (5/9), 0% (0/9)), (p=0.006 and p=0.003), respectively.

The median collagen thickness was 25.1 µm (12.9-46.4) in CCs, 3.91 µm (2.5-6.7) in LCs, and 8.24 µm (6.7-9.1) in MCi. Among CC cases, when collagen thickness was classified as <30 µm and ≥30 µm, sloughing of the superficial mucosa, vacuolization and mucin loss were higher in patients with a thickness ≥30 µm compared to those with collagen thickness <30 µm (p=0.036, p=0.007, p=0.056).

## DISCUSSION

Microscopic colitis (MC) is a chronic inflammatory bowel disease defined by chronic watery diarrhea with normal colonoscopy findings but with evidence of mucosal inflammatory changes in colonic biopsies. Its incidence is increasing and the quality of life in active MC patients is poor but a proper treatment may help a lot of patients to return to normal life. Biopsy findings are pivotal for the diagnosis of MC, and it is crucial for the pathologist to be aware of MC and MCi’s diagnostic clues (7,11,13). This study provides detailed clinical and histopathological findings in colonic biopsies obtained from patients diagnosed with MC and MCi.

All patients had diarrhea at the time of MC diagnosis and the diagnosis was based on both clinical and pathological findings. Typically, MC is a disease of the elderly, with an average age of approximately 65 years. However, given that 25% of MC patients may present younger than 45 years, young patients with chronic diarrhea should also be evaluated for the disease There is marked female predominancy which is less notable in LC in comparison to CC (6,8,11,16). The present study showed female predominance in CC cases also but there was a distinct male predominance in LC and MCi cases. The mean ages of our CC, LC and MCi patients were 59 (22-87), 51 (27-83) and 46 (24-72) years, respectively. The age distribution in our series is consistent with the literature, but the mean age at diagnosis is lower than the literature findings that report the mean age of patients with CC and LC between 64-68 and 51-59 years, respectively (17–20). However, in another Turkish population study, the mean age of diagnosis of LC and CC was 45 (range: 27-68) and 60 years (range: 54-65 years) similar to our findings (21). The number of patients with diagnosis of MC is increasing in Western, Eastern, as well as Asian countries such as Japan and Korea. A possible explanation for the increased frequency of MC might be the increased use of colonoscopy during work-up investigation of chronic diarrhea patients (22). Smoking might be the culprit for the persistence of MC or it could amplify the risk of developing MC at younger ages (23,24). On the other hand, it is shown that the risk of MC might differ among ethnic groups (22). The contrary findings in age and sex distribution in the present study may also support ethnic factors playing a role in the development of the disease.

The etiology of MC is unclear but is probably multifactorial with contributing factors such as infection, smoking, drugs, mucosal immunopathology, dysregulated collagen metabolism (in CC), and genetics (6,7). According to the literature, 20 to 60% of patients with lymphocytic colitis and 17 to 40% of patients with collagenous colitis have co-existent autoimmune diseases, such as rheumatoid arthritis, collagen vascular diseases, or thyroid disorders, and there is also a strong association with celiac disease (1). One of the striking findings of our study was that 29% of LC cases had concomitant celiac disease, and PPIs and NSAIDs were the most commonly used drugs in terms of drug use history. Eight out of 9 patients with a history of PPI use had CC. PPIs are shown to alter intestinal microbiota and induce acid suppression along with increasing intercellular permeability, which may contribute to disease given the known tight junction dysfunction in MC (7).

Endoscopically normal colon with characteristic histopathological findings form the diagnostic cornerstone in MC. Histological key features are essential for the diagnosis, for differentiating the 2 major subtypes -LC and CC- and to avoid missing the diagnosis in MCi cases that show only subtle changes (1,7). In MC, the morphologic findings may be patchy and not continuous. To achieve an accurate diagnosis, biopsies of the right and left colon are recommended; if only the left colon is sampled, the diagnosis of MC could be missed in up to 40% of the cases (1,7,17–19). Adequate sampling is important and may contribute to the diagnosis of MC. In the present study, all cases had multiple biopsies and the most distinct localization for CC and LC was the right colon (60%, 77%), while for MCi it was left colon (60%).

The cellularity in lamina propria is increased in both MC and LC. We observed lymphoplasmacytosis in the lamina propria at different levels in all CC, LC, MCi cases. The grade of lymphoplasmacytosis was moderate/severe in 54% of CC cases while moderate/severe density was infrequent or absent in non-CC cases (14% of LC, 0% of MCi). The significance of lamina propria inflammation was addressed in a recent study in which the biopsies taken prior to an MC diagnosis frequently showed an increased level of lympho-plasmacellular infiltration (10). Therefore, the presence of lymphoplasmacytosis is an important clue to search for the other diagnostic criteria of MC and MCi while evaluating a biopsy at low power. Cells other than lymphoplasmacytes would be a component of lamina propria in MC cases. One of the striking cell types is eosinophil leukocytes. It is believed that eosinophilia may be evident in CC cases when compared to LC (15,16). It has been reported that a lower number of lamina propria eosinophils can be found in LC than in CC (16). However, there are few reports on this subject, and the increase in eosinophils in the lamina propria is an important and unique finding of our study. This may be explained by the increase in drug use, which is blamed for the etiology of microscopic colitis, causing an increase in eosinophils in the lamina propria. In the present study, 75.7% of CCs had 20 or more eosinophil leukocytes per high power field in the lamina propria, and these rates were lower in LC (57.1%) and MCi (11.1%). The acute inflammation is expected to be focal and mild, and should not predominate within the inflammatory infiltrate, as described earlier (1). Neutrophil leukocytes forming cryptitis were observed in the lamina propria in only 3 (8.1%) of our CC cases, and in none of the LC and MCi cases.

The key histological feature of lymphocytic colitis is >20 IELs /100 enterocytes but an increased number of IEL is also evident in CC but not to the same amount as LC. Most of the time, the increase in the number of intraepithelial lymphocytes is so obvious that there is no need for counting. When there is doubt or the number of IELs is borderline, manual counting should be performed and only the IELs in the intercryptical areas should be taken into account (1). Intraepithelial lymphocytosis (>5 IEL/100 surface epithelial cells) was found to be present in 48% of the patients with collagenous colitis, and a slightly thickened subepithelial collagen band (5-10µm) accompanied 24% of the patients with lymphocytic colitis (19). The thickness of the collagen is not the same in different areas, and should be assessed only in well-oriented sections. In CC, the thickness of the collagen usually ranges from 10 to 30 µm (16). In our study, in 97.3% of CCs, IEL was in the range of 5-20, and in 37.8% in the range of 10-20 µm. Further subepithelial collagen band thickness in the range of 5-10 µm in 28% of LC cases. IEL was in the range of 10-20 in one of our MCi cases that we commented as lymphocytic type of incomplete microscopic colitis. A high IEL number despite under 20/100 enterocytes may connect significant clinical and histological overlap between lymphocytic and collagenous colitis and suggests that these are two histological presentations of the same disease entity (1). In our study, Masson Trichrome stain was applied to all CC and MCi cases, and 86% (6/7) of LC cases, while the CD3 stain was applied to 57% (4/7) of LC cases. As per recent guidelines (1,6,10), histological diagnosis is made on H&E sections but an immunohistochemical stain (CD3) to demonstrate T-lymphocytes in LC and LCI and connective tissue stain to reveal the subepithelial collagen band in CC and CCi may be used if there is any doubt.

Damage of the surface epithelium is an important diagnostic clue, especially in CC cases. In our study, epithelial damage characterized by sloughing of the superficial mucosa and vacuolization was more striking in CC cases than LC and MCi cases, as reported in the literature. However, flattening was observed more frequently in LC cases, which is contrary to the literature (1). Epithelial damage with vacuolization and mucin loss were significantly lower in MCis that show less characteristic features than MC.

According to the literature, the degree of inflammation does not correlate with the thickness of the collagen deposit (15). We found that epithelial damage characterized by sloughing of the superficial mucosa, vacuolization and mucin loss were more common in patients with a thickness ≥30 µm compared to those with collagen thickness <30 µm. Entrapped erythrocytes in subepithelial collagen were observed in 91.9% and cells other than erythrocytes were observed in 54.1% of CC cases. This feature was not striking in non-CC cases and could be used as a diagnostic clue in the differential diagnosis of these entities.

Paneth cells are normally present in the right colon, and when paneth cells are seen in the left colon and rectum their presence indicates previous mucosal injury. Left-sided paneth cells are frequently associated with ulcerative colitis (UC), but they are not particularly specific. It is speculated that paneth cell metaplasia may be a marker of CC which is more refractory to therapy (16,20). We observed paneth cell metaplasia in 16% of CC cases located in segments other than the cecum/ascending colon but this feature was not present in the LC and MCi cases.

We noticed that inflammatory bowel disease (6%) was one of the concominant diseases in our study group. In some small series of patients, microscopic colitis and inflammatory bowel disease have been diagnosed at different times which lead to the speculation that MC and inflammatory bowel disease might be related to each other and could represent end points of a spectrum of the same disorder (1). In contrast to this opinion, there are studies that claimed no obvious relation between MC and UC or Crohn’s disease (9). Further studies are required to find out if any relationship exists between these two entities.

The differential diagnosis of MC mainly includes NSAIDs damage, acute infectious colitis, ischemic colitis, and inflammatory bowel disease. In inflammatory bowel disease there is a presentation of a younger patient profile with bloody diarrhea and abnormal endoscopy. Acute inflammation, cryptitis, crypt abscesses, marked architectural distortion, and basal lymphoplasmacytosis are also seen. In acute infectious colitis, there is diffuse, marked polymorphonuclear leukocytes (PMN) and infiltration of crypts and lamina propria, and there is no IEL and increase in subepithelial collagen band thickness. In NSAIDs damage ischemia-like changes, lamina propria hyalinization, focal active colitis, focal cryptitis, and mild increase in IELs are seen. When separating LC and CC from each other, in lymphocyctic colitis there is greater surface/crypt IELs than CC and there is no thickening of subepithelial collagen (15).

In this study, clinical and diagnostic histopathologic parameters have been analyzed for MC and MCi in detail. The limitations of our study are the retrospective design, and the lack of regular clinical and endoscopic follow-up. However, this is one of the largest series as an original article reporting detailed clinical and histopathological characteristics of MC and MCi cases in the Turkish population. One of the striking points of our study was that 29% of LC cases had concomitant celiac disease, and in terms of drug use history PPI and NSAIDs were the most commonly used drugs. The age distribution in our series is consistent with the literature, but is lower than the general average, and contrary to the literature a distinct male predominance in LC and MCi cases were observed. The different findings in age and sex in the present study may suggest the ethnic factors’ role in the development of the disease. Another important finding of our study was increased number of eosinophils in the lamina propria, which may be explained by the increase use of drugs. The presence of overt lymphoplasmacytosis, increased eosinophils, and epithelial damage should alert the pathologist for the diagnosis of complete microscopic colitis. Collagenous colitis may also demonstrate intraepithelial lymphocytosis although not as much as LC. Given that microscopic colitis is a common treatable cause of chronic diarrhoea, awareness of the aforementioned histopathological features is utmost importance for accurate diagnosis and also to avoid missing hidden-incomplete cases. The constellation of clinical and histological findings should enable the pathologist to reach the accurate diagnosis in most cases.

## Conflict of Interest

The authors declare no conflict of interest.
